# Daily Carnosine and Anserine Supplementation Alters Verbal Episodic Memory and Resting State Network Connectivity in Healthy Elderly Adults

**DOI:** 10.3389/fnagi.2015.00219

**Published:** 2015-11-27

**Authors:** Jaroslav Rokicki, Lucia Li, Etsuko Imabayashi, Jun Kaneko, Tatsuhiro Hisatsune, Hiroshi Matsuda

**Affiliations:** ^1^Integrative Brain Imaging Center, National Center of Neurology and PsychiatryTokyo, Japan; ^2^Faculty of Economics-Informatics, University of BialystokVilnius, Lithuania; ^3^Department of Medicine, Faculty of Medicine, Imperial College LondonLondon, UK; ^4^Department of Integrated Biosciences, Graduate School of Frontier Sciences, The University of TokyoTokyo, Japan

**Keywords:** default mode network, resting-state fMRI, carnosine, anserine, memory, aging, connectivity, elderly

## Abstract

Carnosine and anserine are strong antioxidants, previously demonstrated to reduce cognitive decline in animal studies. We aimed to investigate their cognitive and neurophysiological effects, using functional MRI, on humans. Thirty-one healthy participants (age 40–78, 10 male/21 female) were recruited to a double-blind placebo-controlled study. Participants were assigned to twice-daily doses of imidazole dipeptide formula (*n* = 14), containing 500 mg (carnosine/anserine, ratio 1/3) or an identical placebo (*n* = 17). Functional MRI and neuropsychological assessments were carried out at baseline and after 3 months of supplementation. We analyzed resting state functional connectivity with the FSL fMRI analysis package. There were no differences in neuropsychological scores between the groups at baseline. After 3 months of supplementation, the carnosine/anserine group had better verbal episodic memory performance and decreased connectivity in the default mode network, the posterior cingulate cortex and the right fronto parietal network, as compared with the placebo group. Furthermore, there was a correlation between the extents of cognitive and neuroimaging changes. These results suggest that daily carnosine/anserine supplementation can impact cognitive function and that network connectivity changes are associated with its effects.

## 1. Introduction

We live in a world with an aging population. The number of people aged 60 and over is projected to double as a proportion of the global population, from 7% in 2000 to 16% in 2050, taking the number of people aged 60 years and over to 2 billion (Nations, 2013).

Aging is a risk factor for most common neurodegenerative disease, for example Alzheimer's disease (AD; Hebert et al., 2003). Moreover, advancing age is associated with motor and cognitive decline, including in working and episodic memory formation and capacity (Park and Reuter-Lorenz, 2009; van Geldorp et al., 2015).

Nutrition can influence brain function (Bowman et al., 2012; Cooper, 2014). Carnosine is a dietary compound, which has been investigated for its neuronal protection properties in animal and human studies. Carnosine is a strong antioxidant and an endogenous dipeptide synthesized mainly in the skeletal muscle of vertebrates. In bird muscle, another analogs dipeptide, anserine is synthesized. Carnosine and anserine can suppress zinc induced neurotoxicity, inhibit production of oxygen free-radicals and suppress protein glycation, all thought to be important components of neurodegeneration (Hipkiss, 2007; Boldyrev et al., 2013).

In a transgenic mouse model of AD, disease progression was reduced when mice were supplemented with dietary carnosine, compared to mice that had received placebo. This effect was seen both in contextual and cued memory. Moreover, senile plaques did not form in the hippocampus (HC; Herculano et al., 2013). Indeed, Hipkiss and colleagues suggest that carnosine might have therapeutic potential in AD and other neurodegenerative disorders (Hipkiss, 2007).

In this study, we aimed to investigate the cognitive and neurophysiological effect of daily carnosine/anserine supplementation in a healthy elderly population. Resting state functional MRI (rsfMRI) is acquired by performing fMRI on participants asked to lie quietly and think of nothing in particular and assesses brain activity in the absence of specific task demands (Biswal et al., 1995). Resting state functional connectivity (RSFC) provides details about the underlying brain organization (Fox et al., 2007). Several networks of brain regions have been consistently shown to be active during this state and have been termed “resting state networks” (Fox et al., 2007; Smith et al., 2009; Buckner et al., 2013).

The default mode network (DMN) is an important resting state network, which typically shows high activity during rest and is deactivated during task performance (Fox et al., 2007; Buckner et al., 2013). Alterations in its activity or functional connectivity have been found in many disease states characterized by disturbed cognitive or psychological function (Dennis and Thompson, 2014). We investigated the effect of daily carnosine/anserine supplementation on DMN connectivity. Additionally, to investigate this network in more detail we also studied the connectivity of the posterior cingulate cortex (PCC) region, which serves as a critical node of DMN (Zhang et al., 2010).

The Right Fronto-Parietal Network (RFPN) and Left Fronto-Parietal Network (LFPN) are two strongly lateralized maps and have broad involvement in many cognitive functions, including language and memory (McKiernan et al., 2003; Dhanjal and Wise, 2014; Hearne et al., 2015). We also chose to investigate the effect of carnosine/anserine on RSFC of the hippocampus because of its important role in learning, memory formation and normal aging (VanGuilder and Freeman, 2011).

We conducted a double-blind placebo-controlled study to investigate the effect of daily carnosine/anserine supplementation on the cognitive function and RSFC of healthy older adults. We hypothesized that:

1. Carnosine/anserine will ameliorate deterioration of memory function in healthy elderly adults.
2. Carnosine/anserine will alter the connectivity of resting state networks.
3. The extent to which carnosine/anserine affects RSFC of key resting state networks will be correlated with the extent to which carnosine/anserine affects cognitive outcomes.

## 2. Materials and methods

### 2.1. Participants

Thirty-one healthy participants (age 42–78, IQR = 58–72, 10 male/21 female) were recruited from June 2012 to November 2013 from the Tokyo metropolitan area. Each participant gave written informed consent to participate in the study. The study was approved by the Ethics Committee of the University of Tokyo and by the Ethics Committee of the National Center of Neurology and Psychiatry. Exclusion criteria were:

1. Those who had a pre-existing neuropsychiatric disorder or head injury.
2. Having focal lesions, such as a brain tumor or cerebral infarction as detected on baseline MRI scan.
3. Having contraindications to MRI.
4. Subjects with Mini-mental state examination (MMSE) score below 24 (Folstein et al., 1975).

Participants were assigned to twice-daily doses of imidazole dipeptide formula (*n* = 14), 500 mg in total (carnosine/anserine=1/3) derived from chicken meat (produced by *NH foods Ltd., Japan*), or an identical placebo (*n* = 17) in a double-blinded study. The groups were matched according to age and gender (Table 1).

**Table 1:** **Participants demographics**.

	**Car/ans**	**CI**	**Placebo**	**CI**	***p***
*n*	14		17		
Age	61.4	(56.5, 66.4)	65.5	(61.9, 69.2)	0.20
Sex (male/female)	5/9		5/12		0.72
MMSE	28.2	(27.6, 28.2)	29.1	(28.5, 29.6)	0.07
YoE	14.5	(13.7, 15.4)	14.9	(13.8, 15.9)	0.64

*Data are presented as mean, followed by 95% confidence intervals (CI). Car/ans, carnosine/anserine; MMSE, mini-mental state exam; YoE, years of education*.

### 2.2. Cognitive tests

Three kinds of psychological cognitive tests were performed: the Alzheimer's Disease Assessment Scale-Cognition (ADAS-Jcog), Wechsler Memory Scale Logical Memory 1&2 (WMS-LM1&2), and Beck Depression Inventory (BDI).

All used tests were translated into Japanese. The WMS-LM1&2 were used to assess logical and episodic memory for immediate recall and delayed recall respectively. The ADAS-J cog (Alzheimer's Disease Assessment Scale) is the Japanese version of cognitive subscale of ADAS, and was used to evaluate changes in cognitive function over time (Mohs et al., 1983; Homma et al., 1992, 2000). Mood and subjective states were assessed by Japanese version of Beck Depression Inventory (Beck et al., 1996; Kojima et al., 2002).

### 2.3. Data acquisition

MR experiments were performed in a 3T scanner (Siemens, MAGNETOM Verio 3.0T), located at the National Center of Neurology and Psychiatry Facility (Tokyo, Japan). All data were acquired using a 32-channel phased array head coil. MRI data of volunteers were collected at two time points: pre-supplementation (baseline) and the post-supplementation (3 month follow up). In the scanner, headgear and ear plugs were used to limit head motion and reduce scanner noise. rsfMRI scans were acquired using gradient-echo echo-planar sequence with repetition time TR = 3000 ms; echo time TE = 30 ms; flip angle = 80°; with 48 axial slices; slice thickness being 3.3 mm and no gap; each slice consisted of 64 × 64 voxels, resulting in 3.30 × 3.31 × 3.31 voxel dimension. Whole brain high resolution T1-weighted anatomical scan was acquired for registration purposes using magnetization prepared rapid gradient echo (MP-RAGE) sequence with following parameters: TR = 1900 ms, TE = 2.52 ms, TI = 900 ms, flip angle = 9°, field of view = 250 × 250 mm, acquisition matrix = 192 × 256, 256 sagittal slices, slice thickness = 1.0 mm, slice gap = 0 mm, axial slice number = 192, voxel dimension = 1.00 × 0.98 × 0.98. All subjects underwent 7 min (140 images) of scanning and were instructed to remain awake and think of nothing particular with their eyes open.

### 2.4. Data preprocessing

Structural data preprocessing consisted of removal of non-brain tissue and celebro spinal fluid by using Statistical Parametric Mapping software package (SPM12).

rsfMRI data processing was carried out using FEAT (FMRI Expert Analysis Tool) Version 6.00, part of FSL (FMRIB's Software Library, www.fmrib.ox.ac.uk/fsl). Registration to high resolution structural and/or standard space images was carried out using FLIRT (Jenkinson and Smith, 2001; Jenkinson et al., 2002).

Initial preprocessing steps included deleting first 3 volumes of each fMRI series, to allow magnetic field to reach a steady state, motion correction, spatial smoothing using 6 mm full-width-at-half-maximum Gaussian kernel, and high-pass temporal filtering with cut-off frequency being set at 0.01 Hz. Functional images were co-registered to high resolution T1-weighted images by means of boundary-based registration (Greve and Fischl, 2009). Single-session independent component analysis (ICA) was performed by MELODIC, to decompose a single subject 4D dataset into a set of spatial and temporal components.

Subsequently the auto-classification of artefactual ICA spatial components was performed, to remove noise components from the 4D fMRI data by using FIX (Griffanti et al., 2014; Salimi-Khorshidi et al., 2014). FIX was trained using data from 32 subjects randomly selected from the same study (each subject was scanned twice, therefore there were 62 scans available). *Signal* vs. *Noise* ICA components were identified by LL and JR by visual inspection, based on procedures described previously. These procedures rely on examining the frequency spectra of timeseries, pattern of timeseries, sinus co-activation, spike identification and correspondence of activated areas to anatomical regions (Kelly et al., 2010).

Cleaned functional data were registered using 12 degrees-of-freedom to standard space (Montreal Neurological Institute atlas) and resampled to 4 × 4 × 4 mm resolution. Afterwards, data were temporally concatenated across all subjects to create a single 4-dimensional dataset and inputed to group-ICA analysis.

**Figure 1:**
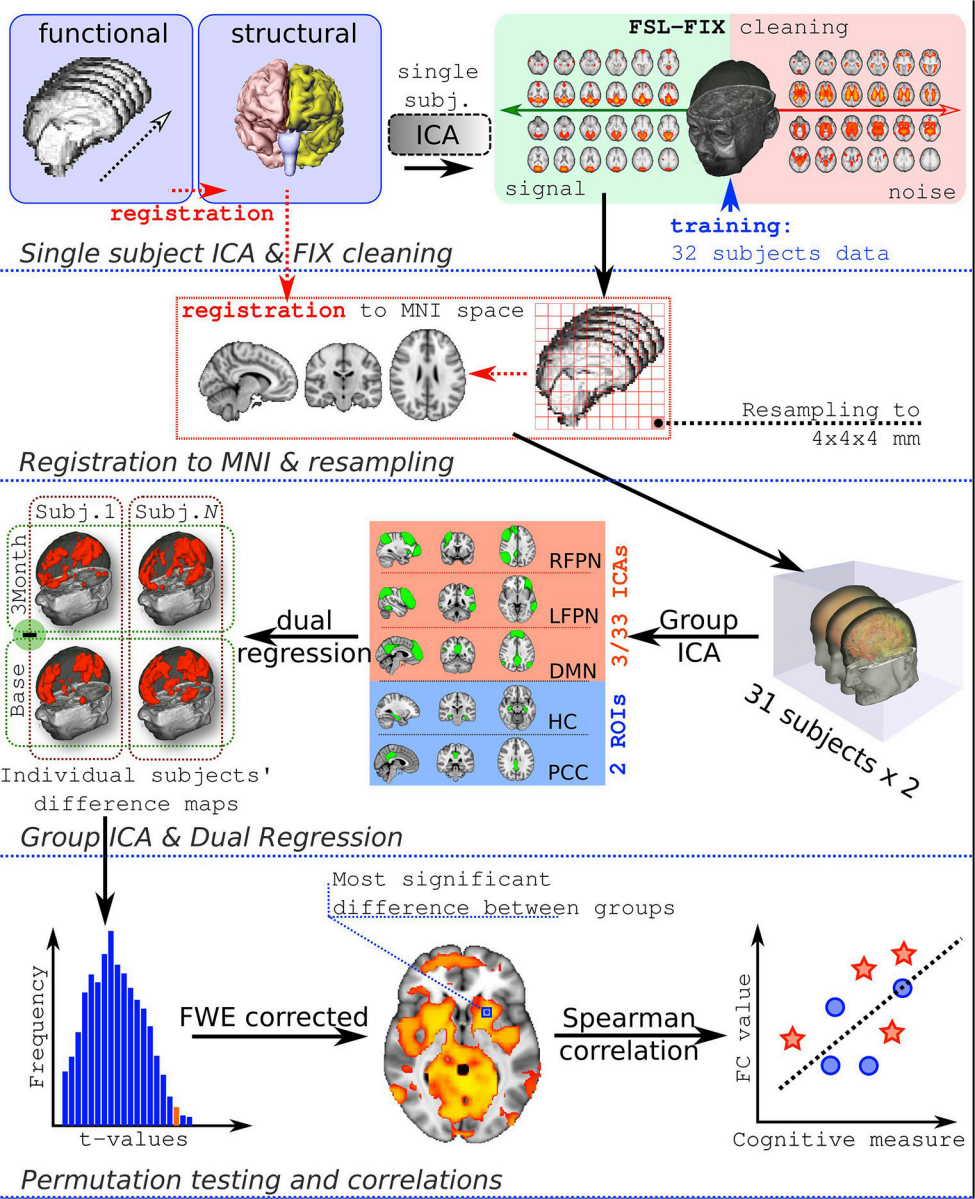
**Analysis workflow**. FC, functional connectivity.

The full workflow is presented in Figure 1.

### 2.5. Motion quality control

We assessed head motion translation and rotation separately using the formula (Liu et al., 2008):

(1) $$\begin{array}{l} \frac{1}{1-M}\overset{M}{\underset{i=2}{\sum }}\sqrt{{\left(x_{i}-x_{i-1}\right)}^{2}+{\left(y_{i}-y_{i-1}\right)}^{2}+{\left(z_{i}-z_{i-1}\right)}^{2}} \end{array}$$

The results showed that the two groups had no significant differences in rotational and translational head motion. Moreover, there was no significant differences between baseline and follow-up.

No subjects were excluded due to excessive motion (exclusion criteria were average translation >0.3 mm or average rotation>0.3°). Detailed information on motion is presented in Table 2.

**Table 2:** **Average group head motion assessed separately for translation and rotation**.

**Group**	**Translation, mm**	**Rotation, deg**	***p*-value**
	**BASELINE**		
Carnosine/anserine	0.042	0.033	0.57
Placebo	0.045	0.030	0.44
	**3-MONTH FOLLOW-UP**		
Carnosine/anserine	0.047	0.031	0.52
Placebo	0.053	0.035	0.44
*p*-value	0.26	0.81	

*Last column shows p-value of two sample t-test between carnosine/anserine and placebo groups. Last line shows the p-value of two sample t-test between baseline and 3-month follow up. No significant differences are present*.

Spike-like movements and other movement or physiological artifacts were removed using FSL-FIX software.

### 2.6. Component selection and connectivity analysis

Groups spatial ICA was conducted on all 31 subjects and both timepoints combined together to ensure that the resulting components had similar resting state fluctuations in all the participants at all timepoints of the study. Dimension estimation was done automatically, resulting in 33 spatial components. The components were identified by LL and JR according to Smith et al. (2009).

**Figure 2:**
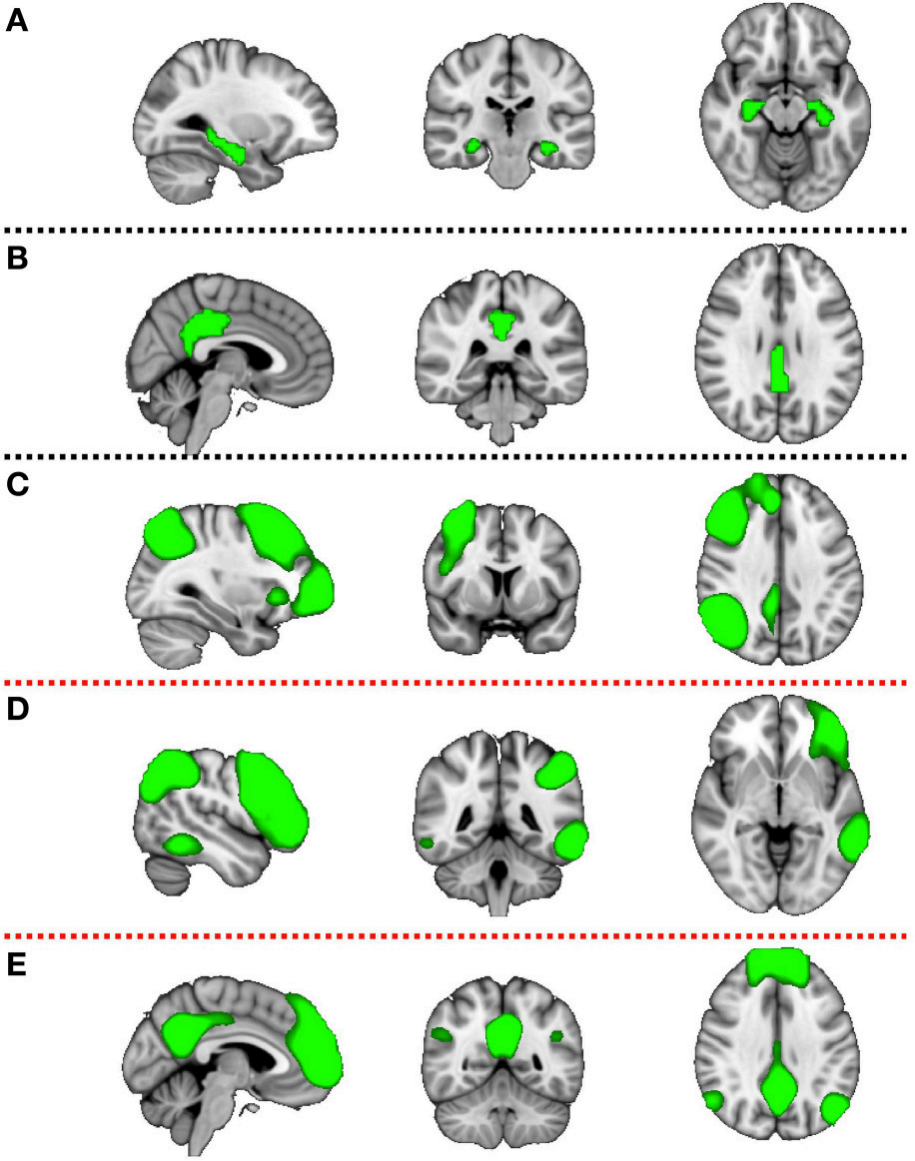
**Five masks used in further ***dual-regression*** analysis**. The figure shows the three most informative orthogonal slices for each mask. Green colored mask is scaled to fit and superimposed onto the 1-mm standard MNI152 standard space template image. Masks are: **(A)** left and right hippocampus (Montreal Neurological institute (MNI) coordinates *x* = 3, *y* = −35, *z* = 28), **(B)** posterior cingulate cortex (*x* = −28, *y* = −24, *z* = −14), **(C)** right fronto parietal network (*x* = 32, *y* = 7, *z* = 35), **(D)** left fronto parietal network (*x* = −47, *y* = −44, *z* = −4), **(E)** default mode network (*x* = −4, *y* = −53, *z* = −35). First two masks **(A,B)** are binary masks, while last three **(C–E)** are components produced by the group ICA decomposition of rsfMRI data and converted to *Z* statistic images via normalized mixture-model fit, and thresholded at arbitrary threshold *Z* = 3 for visualization purposes.

Components of interest (DMN, RFPN, and LFPN) were identified visually and additionally PCC and HC ROIs were constructed (Figure 2). The HC mask was created using the Harvard-Oxford Subcortical Atlas. The PCC mask was prepared using the Harvard-Oxford Cortical Atlas. Both masks were thresholded at 50% probability and binirized. This set of spatial maps from the group-average analysis and ROIs was used to generate subject-specific versions of the spatial maps, and associated timeseries, using dual regression (Filippini et al., 2009). First, each spatial map was used as a spatial regressor in a single variable regression in the preprocessed functional scan. This produced a timeseries for the inputted spatial map. Next, the timeseries was regressed (as temporal regressors in a single variable regression into the same functional scan). Both steps were repeated for each subject, at both timepoints (pre and post supplementation), resulting in a set of subject-specific spatial maps (“connectivity maps”) at each timepoint, one per group-level spatial map. The fslmaths command was used to find the difference in the connectivity maps pre and post supplementation for each subject (carnosine/anserine and placebo). This gave the change in connectivity over 3 months for carnosine/anserine and placebo. Finally, we tested for a group (carnosine/answer vs. placebo) difference in this change in connectivity, using FSL's randomize permutation-testing tool. This procedure resulted in testing for Group × Time interaction in data. All imaging data were corrected using family-wise error (FWE) for multiple comparisons along with threshold free subject enhancement using a significance level *p* < 0.05 (Smith and Nichols, 2009; Winkler et al., 2014). Additionally, because we used multiple maps rather than concentrate on a single map some further correction was performed. Since we selected 5 masks as being of potential interest and two contrasts were tested for each map (Placebo > Carnosine/anserine and Carnosine/anserine > Placebo) the final significance threshold was adjusted to *p* = 0.05∕(5 × 2) = 0.005.

Spearman's correlation was performed to investigate the correlation between functional connectivity changes as assessed by fMRI and cognitive score changes using packages hmisc (for full correlation) and ppcor (for partial correlation) in R (http://www.r-project.org/). The voxel (4 × 4 × 4 mm) with the highest significance value on the family-wise error corrected comparison maps was identified (**Table 4**). These were used to extract a connectivity value from the spatial maps produced from the second stage of the dual regression procedure of each participant at both time points (baseline and 3 months). The change in connectivity within each person then was calculated as the difference between the connectivity values at the two different timepoints.

For the result visualization we used FSLView and BrainVis softwares. For volume-to-surface mapping we used “Average Voxel” option, that is assign the vertex with average value of the voxel and its neighbors in volume that is nearest to it (Xia et al., 2013).

## 3. Results

### 3.1. Cognitive results

There were no significant differences between the carnosine/anserine and placebo groups at the baseline in WMS-LM2, BDI, and ADAS-Cog. After 3 months supplementation, the decline in verbal episodic memory score as measured with WMS-LM2 was significantly less in the carnosine/anserine group (−0.29±3.45) than in the placebo group (−3.06±3.86) (*p* = 0.046, Cohen's *d* = 0.75). There were no differences between the carnosine/anserine and placebo groups in BDI and ADAS-Cog score. The cognitive results are summarized in Table 3.

**Table 3:** **Neuropsychological test outcomes**.

	**Car/ans**	**CI**	**Placebo**	**CI**	***p***
	**BASELINE**				
WMS-LM1	13.2	(10.7, 15.7)	14.4	(12.7, 16.1)	0.43
WMS-LM2	12.1	(9.9, 14.4)	13.5	(12.2, 14.9)	0.30
Adas-JCog	6.6	(4.9, 8.3)	5.8	(4.1, 7.4)	0.51
BDI	7.7	(4.1, 11.3)	9.4	(6.0, 12.8)	0.51
**3 MONTH FOLLOW UP**					
WMS-LM1	12.2	(10.1, 14.3)	12.1	(10.3, 14.0)	0.95
WMS-LM2	11.9	(9.9, 13.8)	10.5	(8.4, 12.5)	0.35
Adas-JCog	6.4	(5.0, 7.8)	5.8	(3.8, 7.8)	0.66
BDI	6.4	(2.9, 9.8)	6.2	(3.8, 8.5)	0.93
**TIME** × **GROUP INTERACTION**					
WMS-LM1	−1.0	(−3.0, 1.0)	−2.3	(−3.6, −0.9)	0.29
WMS-LM2	−0.3	(−2.1, 1.5)	−3.1	(−4.9, −1.2)	0.046^*^
Adas-JCog	−0.2	(−1.2, 0.8)	0.1	(−2.0, 2.1)	0.84
BDI	−1.4	(−4.7, 2.0)	−3.6	(−5.2, −1.3)	0.33

Data are presented as mean, followed by 95% confidence intervals (CI) and significance value. Significant results are marked

* *being significant at 0.05 level. Car/ans, carnosine/anserine subjects*.

### 3.2. Imaging results

There was no any significant differences between the carnosine/anserine and placebo groups at the baseline (Table 4).

**Table 4:** **Minimal family-wise error corrected ***p***-value of the investigated rsfMRI networks for carnosine/anserine (C) group compared against placebo (P) group**.

		**Baseline**		**Time**× **Group**		**MNI Coordinates**	**Brain region**
		**C>P**	**P>C**	**C>P**	**P>C**		
(a)	HC-ROI	0.530	0.222	0.668	0.332		
(b)	PCC-ROI	0.737	0.117	0.998	0.002^*,**^	−6, −54, 4	Left precuneous cortex
(c)	ICA-RFPN	0.641	0.074	0.888	0.003^*,**^	6, −22, 4	Right thalamus
(d)	ICA-LFPN	0.509	0.628	0.927	0.072		
(e)	ICA-DMN	0.818	0.302	0.843	0.010^*^	−18, −34, −8	Left hippocampus

Significant results are marked ^*^ being significant at 0.05 level and ^**^ for being significant after additional Bonferroni correction (p = 0.005).

*First 2 entries of the table are ROIs, while last 3 entries correspond to ICA maps generated from study data. The MNI cooridates (x, y, z) of the voxel where the most significant difference between groups is present, together with region name the voxel belongs too (according to Harvard-Oxford Cortical and Subcortical Structural Atlases) are presented for p < 0.05 resutls only*.

The group which had taken carnosine/anserine showed decreased connectivity for DMN, RFPN, and PCC masks in comparison with the placebo group at *p* < 0.05. If correction for multiple maps and contrasts is used (*p* = 0.005) only two masks survive significance threshold: PCC and RFPN.

**Figure 3:**
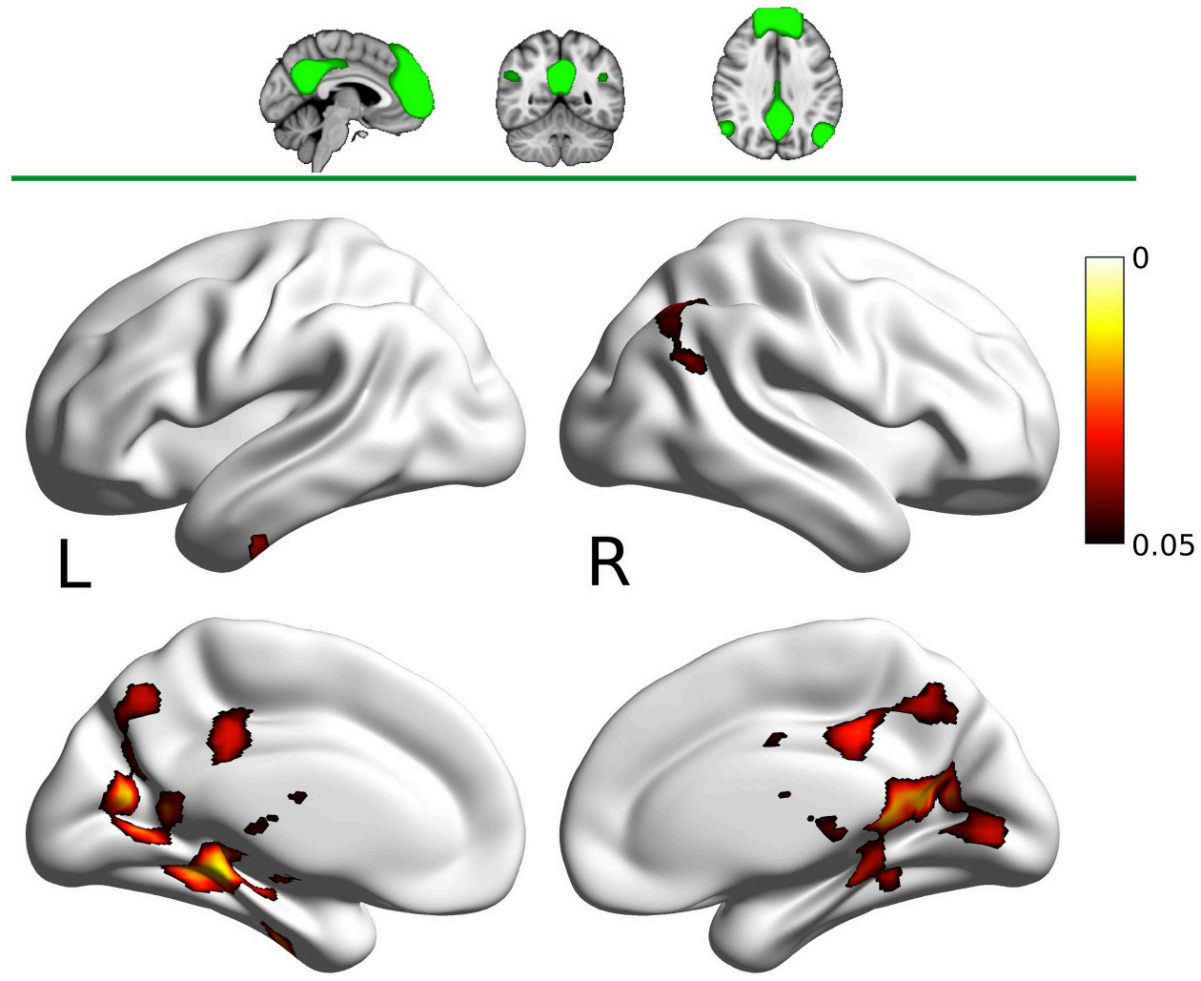
**Results for the default mode network (DMN) mask (two dimensional image on top, with mask shaded in green, centered at ***x*** = −4, ***y*** = −53, ***z*** = −35)**. Warm colors present the cluster of decreased connectivity in carnosine/anserine subjects (multiple comparison FWE corrected, *p* < 0.05). Left (L) and right (R) hemispheres are presented separately.

The DMN map showed decreased connectivity in the carnosine/anserine group, as compared to the placebo group with bilateral thalami, hippocampal areas, putamen and pallidium, precuneus cortex, right lateral occipital cortex and PCC (all at *p* < 0.05; Figure 3).

**Figure 4:**
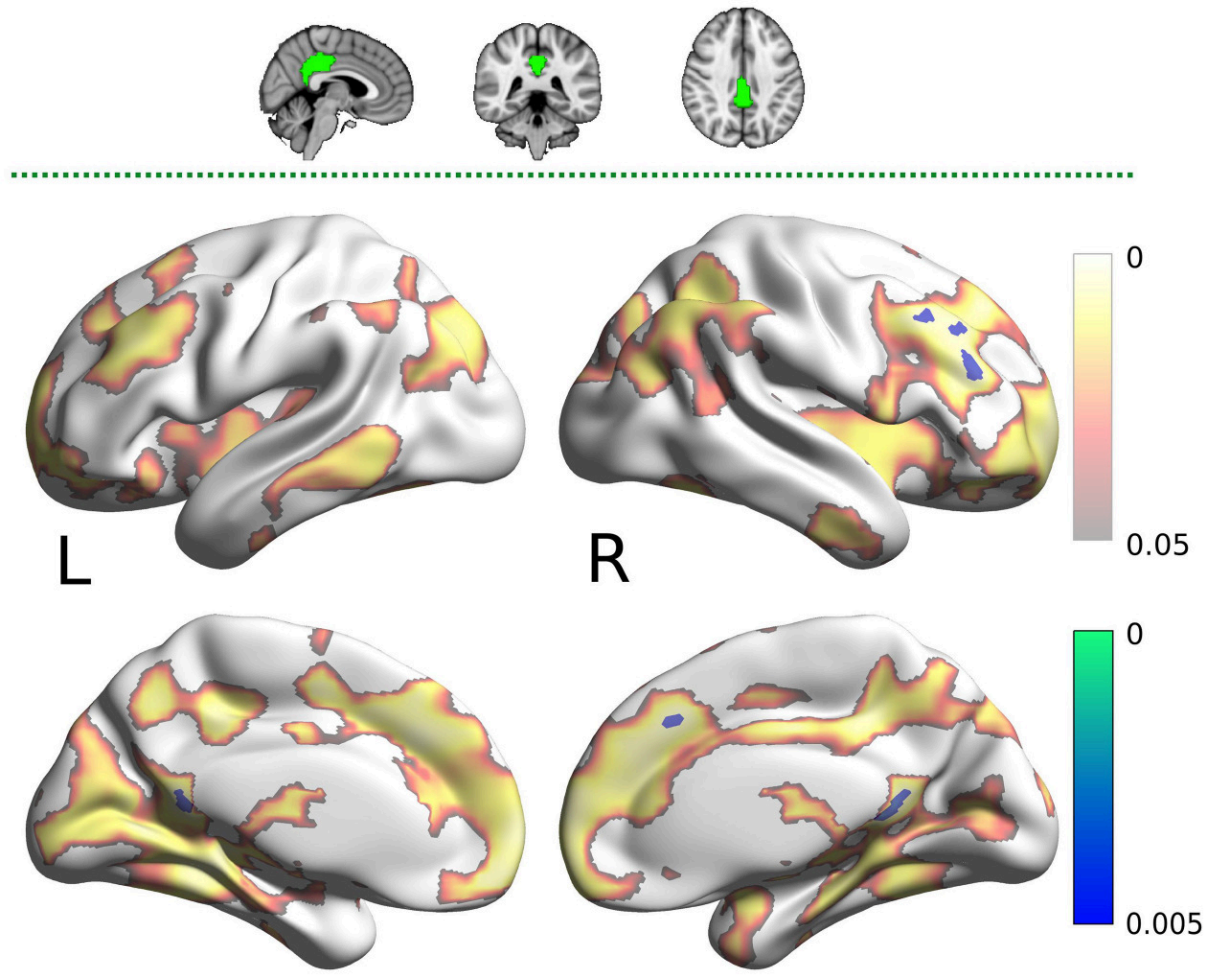
**Results for the posterior cingulate cortex (PCC) mask (two dimensional image on top, with mask shaded in green, centered at ***x*** = −28, ***y*** = −24, ***z*** = −14)**. Warm colors present the cluster of decreased connectivity in carnosine/anserine subjects (multiple comparison FWE corrected, *p* < 0.05). Bluish colors present the areas in which carnosine/anserine subjects have decreased connectivity as compared to placebo with additional Bonferroni correction (*p* < 0.005). Left (L) and right (R) hemispheres are presented separately.

The PCC mask showed decreased connectivity with carnosine/anserine group in the areas similar to DMN (Figure 4), but with significance surviving comparisons for multiple maps and contrasts. These areas include: precuneus cortex, right frontal lobe as well as with other parts of PCC itself (all at *p* < 0.005).

**Figure 5:**
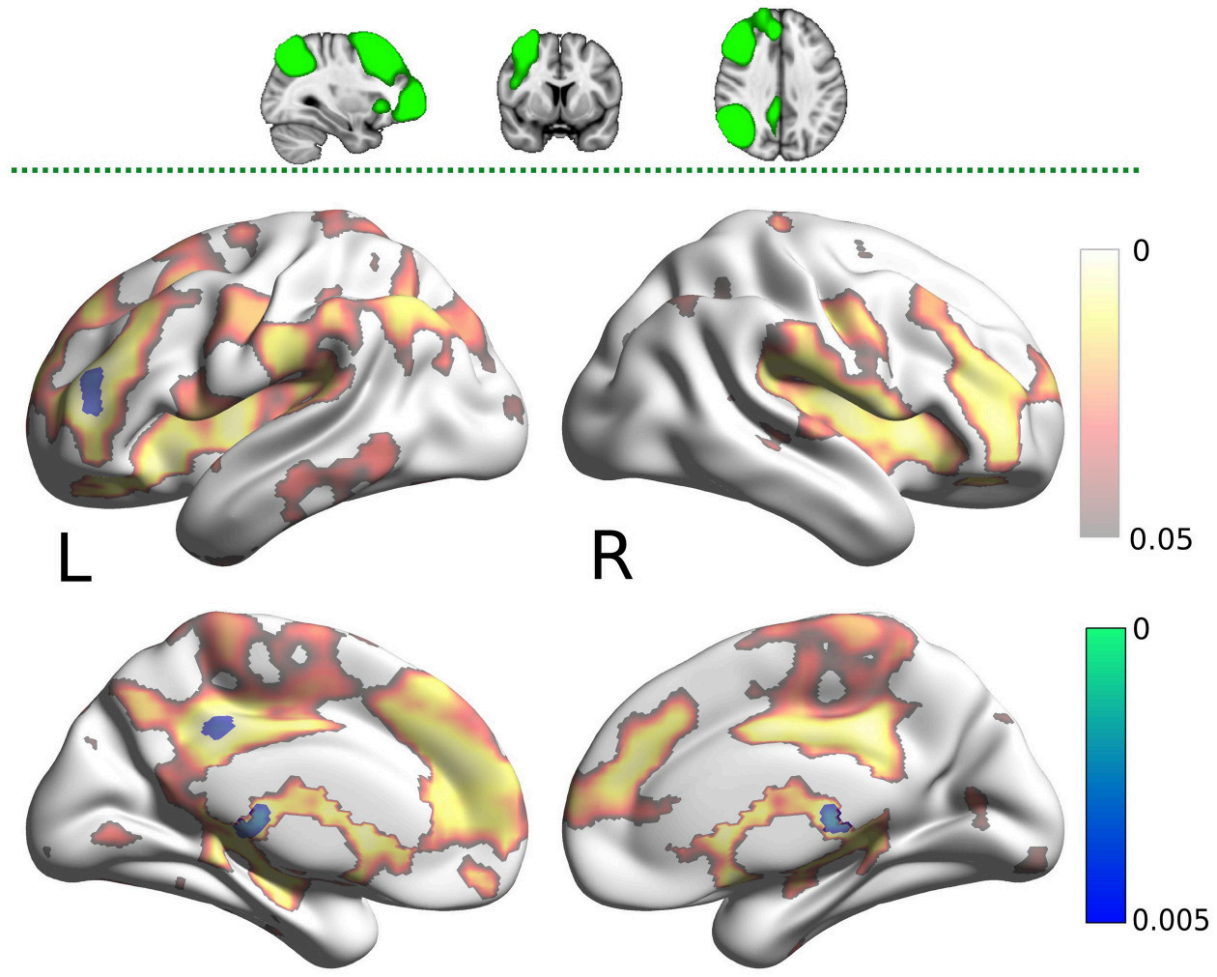
**Results for the right fronto-parietal network (RFPN) mask (two dimensional image on top, with mask shaded in green, centered at ***x*** = 32, ***y*** = 7, ***z*** = 35)**. Warm colors present the cluster of decreased connectivity in carnosine/anserine subjects (multiple comparison FWE corrected, *p* < 0.05). Bluish colors present the areas in which carnosine/anserine subjects have decreased connectivity as compared to placebo with additional Bonferroni correction (*p* < 0.005). Left (L) and right (R) hemispheres are presented separately.

The carnosine/anserine group also showed decreased connectivity between the RFPN (Figure 5) and bilateral thalami and PCC (*p* < 0.005).

There was no significant difference in hippocampal or LFPN connectivity when comparing the carnosine/anserine and placebo groups.

All results are listed in Table 4.

**Figure 6:**
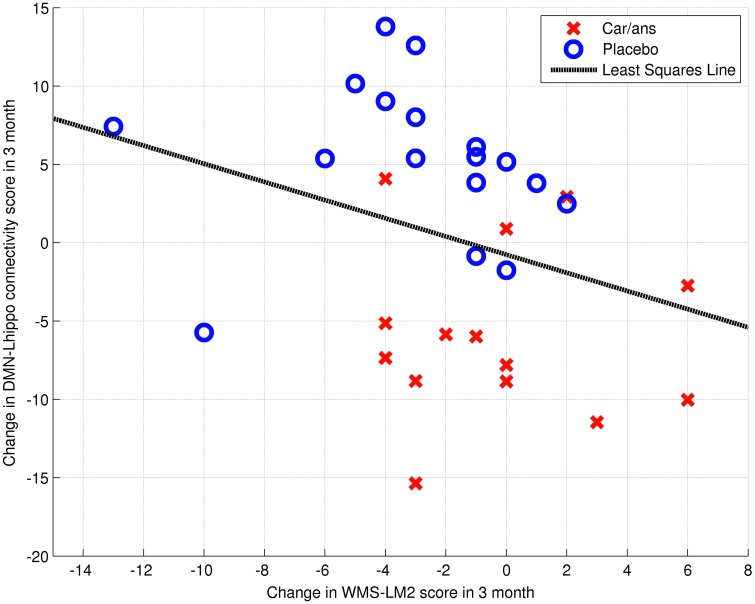
**Dependency between change in WMS-LM2 score and change in connectivity between DMN and Left hippocamus/WM voxel (MNI coordinates: ***x*** = −18, ***y*** = −34, ***z*** = −8; Spearman's ***r*** = 0.446, ***p*** = 0.043)**. Carnosine/anserine (car/ans) subjects are presented with red crosses and placebo subjects with blue circles.

**Figure 7:**
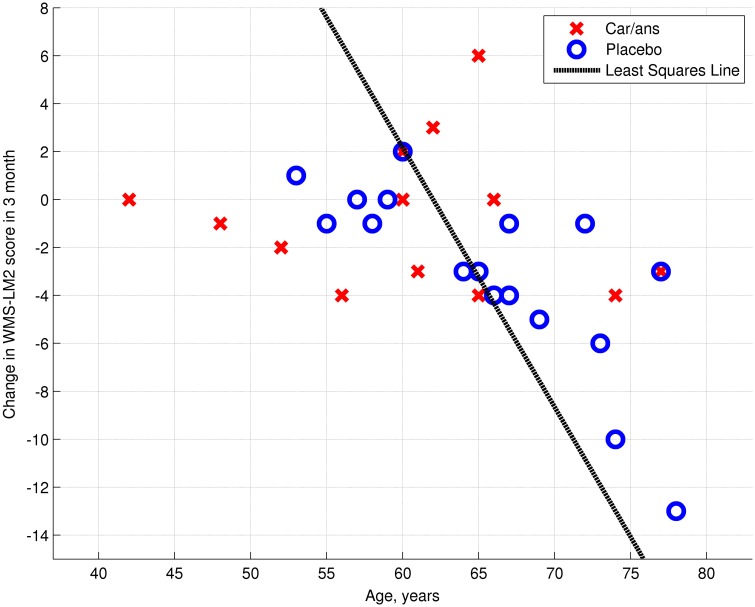
**Dependency between age and change in WMS-LM2 score (Spearman's ***r*** = 0.493, ***p*** = 0.023)**. Carnosine/anserine (car/ans) subjects are presented with red crosses and placebo subjects with blue circles.

The left posterior hippocampus (*x* = −18, *y* = −34, *z* = −8) represented the area where the change in connectivity between the two groups was most significant (Table 4). The extent to which the connectivity between the DMN and left posterior hippocampus decreased was negatively correlated with the decline in WMS-LM2 score (Spearman's *r* = −0.446, *p* = 0.043). That is, a greater decrease in connectivity was associated with less decline in WMS-LM2 score (Figure 6). There was also a correlation between age and change in WMS-LM2 score (Spearman's *r* = −0.493, *p* = 0.023), indicating that older age correlated with greater decline in WMS-LM2 score (Figure 7). There was no correlation between age and change in the DMN-left hippocampus connectivity. To be sure, that the correlation between the change WMS-LM2 score and the change in DMN was not solely driven by subjects' age we calculated partial Spearman's correlation coefficient between these two variables, while controlling for age. The strength of correlation became weaker, but remained significant (Spearman's *r* = −0.391, *p* = 0.024).

## 4. Discussion

We performed an exploratory study of daily carnosine/anserine supplementation on cognitive function and, for the first time to our knowledge, on functional connectivity in healthy elderly subjects. We demonstrate that carnosine/anserine supplementation results in decreased functional connectivity within the DMN, and from the RFPN and DMN to other brain regions. We also found that carnosine/anserine supplementation reduced the decline in WMS-LM2 score over 3 months period, as compared to the placebo group. Furthermore, the extent of change in connectivity between DMN and left posterior hippocampus correlated with WMS-LM2 score change; subjects with lowest decline also had the greatest DMN-hippocampus connectivity decrease.

Previous studies of carnosine/anserine supplementation have found improved cognitive function and physical capacity in animal (Herculano et al., 2013) and elderly human subjects (Szcześniak et al., 2014).

Our study demonstrates that carnosine/anserine supplementation produced decreased connectivity within and between the DMN and widespread brain regions, including subcortical nuclei, hippocampal areas and thalami, as well as decreased connectivity between the RFPN and bilateral thalami. Of note is that although a similar spatial pattern of functional connectivity changes were observed when the DMN and PCC were used as regions of interest in the connectivity analyzes, the changes seen when using the PCC as ROI were more significant. The correlation between functional connectivity changes and WMS-LM2 score change suggests that the neuroimaging changes are, at the very least, a biomarker for the action of carnosine/anserine on verbal episodic memory.

Previous studies have linked decreased DMN connectivity to reduced cognitive performance in healthy aging (Hafkemeijer et al., 2012; Toussaint et al., 2014; Vidal-Piñeiro et al., 2014). DMN-hippocampal connectivity was positively correlated with verbal episodic memory function in healthy older adults (Salami et al., 2014). Meanwhile, increased connectivity within the hippocampi itself is also seen to be correlated with poorer verbal episodic memory performance in older adults (Salami et al., 2014). The DMN also shows load-dependent deactivation as task difficulty increases and failure of this network to deactivate, due to its disconnection with task-related networks, has been associated with poor cognitive function in both healthy individuals and brain injured patients (Sharp et al., 2014).

We can also consider the evidence from neurodegenerative diseases, bearing in mind that the mechanisms underlying neurodegenerative diseases may not be identical to those underlying normal aging. Decreased functional DMN connectivity was linked with reduced cognitive performance in mild cognitive impairment and AD (Agosta et al., 2012; Binnewijzend et al., 2012; Toussaint et al., 2014). Reduced connectivity between the RFPN and various frontal regions has also been noted in AD (Agosta et al., 2012), and reduced connectivity between the thalamus and dorsolateral prefrontal cortices have been seen in mild cognitive impairment (Liang et al., 2011). A recent study examining functional connectivity changes in AD also found evidence of altered connectivity between thalami and regions of the DMN and frontoparietal areas having decreased connectivity in AD and MCI groups compared to controls (Zhou et al., 2013).

On the other hand, it is still not completely clear that decreased connectivity is necessarily detrimental to cognition. For example, increased functional connectivity within the right PCC has been observed in patients with dementia with Lewy body (Kenny et al., 2012). In AD, increased hippocampal connectivity (Kenny et al., 2012) and increased connectivity within frontal regions (Liang et al., 2011) have all been observed. Increased connectivity within the hippocampi is also seen to be correlated with poorer verbal episodic memory performance in older adults (Salami et al., 2014). Of particular interest is the finding that if DMN is fractionated into subsystems and the connectivity within each subsystem is separately analyzed, the anteroposterior system shows decreased functional connectivity in elderly controls, whereas the frontal and parietal subsystems showed increased connectivity (Toussaint et al., 2014). It may be that even within the same network, there are distinct sub-networks and regions showing differing patterns of altered connectivity in states of abnormal cognition.

A possible explanation for our finding of decreased functional connectivity in participants who had taken carnosine/anserine supplementation may be decreased neuroinflammation. Neural trauma is known to cause neuroinflammation of brain (Kumar and Loane, 2012) and chronic inflammation is connected to normal and pathological aging (Chung et al., 2009). American Football athletes, who experience repetitive neural traumas have shown hyperconnectivity as compared to their non-collision counterparts, in the absence of measurable cognitive impairment (Abbas et al., 2014). Abbas and colleagues hypothesize that the hyperconnectivity is a compensatory mechanism allowing for normal cognitive function in the presence of repetitive neural trauma (Abbas et al., 2014).

Neuroinflammation is also associated with normal aging, especially within the hippocampus region (Hein and O'Banion, 2009). Several antioxidants showed ability to help prevent neuroinflammation in rodent studies including caffeine (Ullah et al., 2015) and natural compound isolated from the lapacho tree (Lee et al., 2015). Neurotrauma, which can cause extensive neuroinflammation, is associated with grossly abnormal functional connectivity patterns, including evidence of increased functional connectivity (Bonnelle et al., 2011; Abbas et al., 2014; Sharp et al., 2014).

Moreover, our hypothesis is supported by animal studies which show that carnosine/anserine supplementation can reduce expression of receptors for advanced glycation endproducts in blood vessels in mice. Such expression occurs in the development of many degenerative diseases including AD, as well as microglial activation, mainly caused by neuroinflammation (Herculano et al., 2013).

In summary, we demonstrated that carnosine/anserine supplementation in healthy older adults led to improved performance on a verbal episodic memory task and decreased functional connectivity in PCC and RFPN networks. Moreover, there was correlation between the WMS-LM2 scores and RSFC values suggesting that functional connectivity can be a biomarker of these cognitive benefits.

### 4.1. Limitations

The findings of our study must be interpreted with some limitations in mind. First, this study had a relatively small sample size and short duration of follow-up. Secondly, because this study was set up to investigate the effects of supplementation in elderly adults, we cannot make conclusions about any possible effects of similar supplementation in younger adults. It may be that, in a group that does not experience natural cognitive decline over time, supplementation would not have any discernible cognitive effect. Thirdly, the change in WMS-LM2 score also correlated with age, although the change in DMN connectivity did not correlate with age. This suggests that there is some interaction between supplementation and age, which is not easily distinguishable in our study.

### 4.2. Concluding remarks

This is the first neuroimaging study of the effects of carnosine/anserine supplementation in humans. The results indicate that carnosine/anserine supplementation may have cognitive benefits. Furthermore, changes in resting state functional connectivity may be a potential biomarker these cognitive benefits. An expanded study is currently under way.

## Disclosure

This study was supported by the Scientific Technique Research Promotion Program for Agriculture, Forestry, Fisheries and Food Industry (Grant# 25012A), from the Ministry of Agriculture, Forestry, and Fishery (MAFF), the Government of Japan.

## Author contributions

Guarantors of integrity of entire study: TH; study concepts and study design: TH, HM; data acquisition: EI, TH, JK, HM; data analysis: JR, LL; literature research: LL, JR; statistical analysis: JR, LL; manuscript preparation: JR, LL; data visualization: JR; manuscript final version approval: all authors.

### Conflict of interest statement

NH foods Ltd., company provided us the supplements free of charge but provided no other financial support. The authors declare that the research was conducted in the absence of any commercial or financial relationships that could be construed as a potential conflict of interest.
